# Fingerprinting of volatile profiles of sprouted and unsprouted seeds and flours of *Phaseolus vulgaris* cultivars using HS-SPME/GC-MS

**DOI:** 10.1371/journal.pone.0360936

**Published:** 2026-10-29

**Authors:** Imene Rajhi, Rim Nefissi Ouertani, Guido Flamini, Haythem Mhadhbi

**Affiliations:** 1 Laboratory of Legumes and Sustainable Agrosystems, Centre of Biotechnology of Borj Cedria, Hammam-Lif Tunisia; 2 Laboratory of Plant Molecular Physiology, Centre of Biotechnology of Borj Cedria, Hammam-Lif, Tunisia; 3 Dipartimento di Farmacia, Pisa, Italy; 4 Centro Interdipartimentale di Ricerca “Nutraceutica e Alimentazione per la Salute” Nutrafood, Università di Pisa, Pisa, Italy; Central Food Technological Research Institute CSIR, INDIA

## Abstract

Legumes are a vital source of plant-based protein and bioactive compounds, yet their consumption is often limited by the development of undesirable off-flavors during processing. This study investigated how germination and milling influence the volatile organic compound (VOC) profiles of three *Phaseolus vulgaris* cultivars. VOCs from both unsprouted and sprouted seeds, as well as their corresponding flours, were analyzed using headspace solid-phase microextraction coupled with gas chromatography–mass spectrometry. In total, 79 VOCs were identified in the seeds and 57 VOCs in the flours. These compounds were classified into seven major chemical groups, including terpene- and non-terpene-derived constituents. The results revealed that germination significantly altered the VOC profile, notably increasing non-terpene derivatives, especially aldehydes, while reducing terpene-based compounds, indicating intensified lipid oxidation. Milling further modified the aroma composition by increasing monoterpene hydrocarbons and decreasing certain non-terpene compounds. Multivariate analyses, including principal component analysis and hierarchical clustering, effectively differentiated the bean cultivars and processing treatments, and identified key marker compounds responsible for these variations. *These findings provide a chemical characterization of* VOC *changes induced by germination and milling in Phaseolus vulgaris,* contributing to the development of processing strategies aimed at optimizing the sensory quality and consumer acceptability of legume-based products.

## Introduction

*Fabaceae* family, commonly known as legumes, is the second largest plant family [1]. Legumes represent one of the most important dietary sources of nutrients, particularly in developing countries, where they provide an affordable and sustainable alternative to animal protein [2,3]. Pulses are rich in macronutrients, containing approximately 20–45% protein, up to 60% carbohydrates, and 5–37% dietary fiber [1,4]. In addition to their nutritional value, they possess significant nutraceutical potential, as they are abundant in bioactive compounds that have been associated with protective effects against cancer, cardiovascular diseases, and neurodegenerative disorders [5].

Despite their nutritional benefits, the culinary use of pulses is often limited by undesirable off‑flavors, typically described as beany, grassy, earthy, or hay‑like, which reduce consumer acceptance and limit market expansion. These off‑notes are largely attributed to volatile organic compounds (VOCs) formed through biochemical processes such as the oxidation of unsaturated fatty acids, degradation of free amino acids, and carotenoid breakdown during seed development, storage, and processing (e.g., dehulling, milling, germination) [6]. VOC profiles in legumes are influenced by multiple intrinsic and extrinsic factors, including cultivar or genotype, environmental growing conditions, crop year, and processing methods, all of which contribute to variations in aroma quality and volatile composition [7].

Advances in analytical methodologies, particularly headspace solid‑phase microextraction coupled with gas chromatography–mass spectrometry (HS‑SPME/GC‑MS), have enabled detailed volatile fingerprinting, facilitating the identification of key odor‑active compounds and the discrimination of cultivars and treatments based on their VOC profiles. Fingerprinting of volatile profiles in sprouted and unsprouted seeds and flours of *Phaseolus vulgaris* is essential for understanding how germination and milling modify aroma‑related compounds, guiding strategies to optimize sensory quality and improve flavor traits in bean‑based products. Although VOC profiling has enhanced understanding of off‑flavor origins in legumes, comprehensive data on odor‑active compounds in common beans remain limited, underscoring the need for systematic studies that integrate volatile profiling with processing and genetic influences to improve product quality and consumer acceptance [8,9].

Therefore, the objectives of this study were: (1) to evaluate the impact of germination and milling on the VOC profiles of three *Phaseolus vulgaris* cultivars (Navy, Kidney, and Black beans) using HS-SPME/GC-MS, and (2) to identify key discriminant compounds in raw and germinated seeds and flours through multivariate analysis, including principal component analysis (PCA).

The novelty of this work lies in the combined use of HS-SPME/GC-MS and multivariate analysis to systematically characterize and differentiate the volatile profiles of sprouted and unsprouted common beans and their flours. This integrative approach provides new insights into how processing, specifically germination and milling, modulates aroma compounds, contributing to a deeper understanding of flavor development in legumes.

## Materials and methods

### Chemicals and reagents

A pure reference compounds including heptanal, benzaldehyde, octanal, (E)-2-octenal, decanal, dodecanal, all *n*-hydrocarbons, butyl butyrate, 6-methyl-5-hepten-2-one, naphthalene, acetophenone, phenol, β-pinene, p-cymene, limonene, γ-terpinene, 1,8-cineole, α-terpineol, verbenone, bornyl acetate, longifolene, geranylacetone (E + Z isomers), 1-octanol, 1-decanol, ethyl benzoate, 2-undecanone, α-thujone + β-thujone mixture, and pulegone were purchased from Sigma, Aldrich, Supelco and Merck. These standards were used to confirm compound identities through comparison of retention times and mass spectra.

### Sample preparation and replication

Three *Phaseolus vulgaris* cultivars (Navy, Kidney, and Black beans) were used in this study. Uniform seeds without physical damage were selected and stored at 4°C in opaque aluminium bags until analysis. For each of the three cultivars, a large number of seeds were used to ensure biological replication. Separate batches of seeds were prepared for raw seeds, germinated seeds, raw flour, and germinated flour. This allowed me to account for natural differences between individual seeds and ensure that the results are reliable and reproducible (Fig 1). For GC-MS analysis, each sample was injected three times (technical replicates). The relative peak areas obtained from the three injections were averaged, and the mean values were used for all statistical analyses, including PCA and HCA.

**Fig 1:**
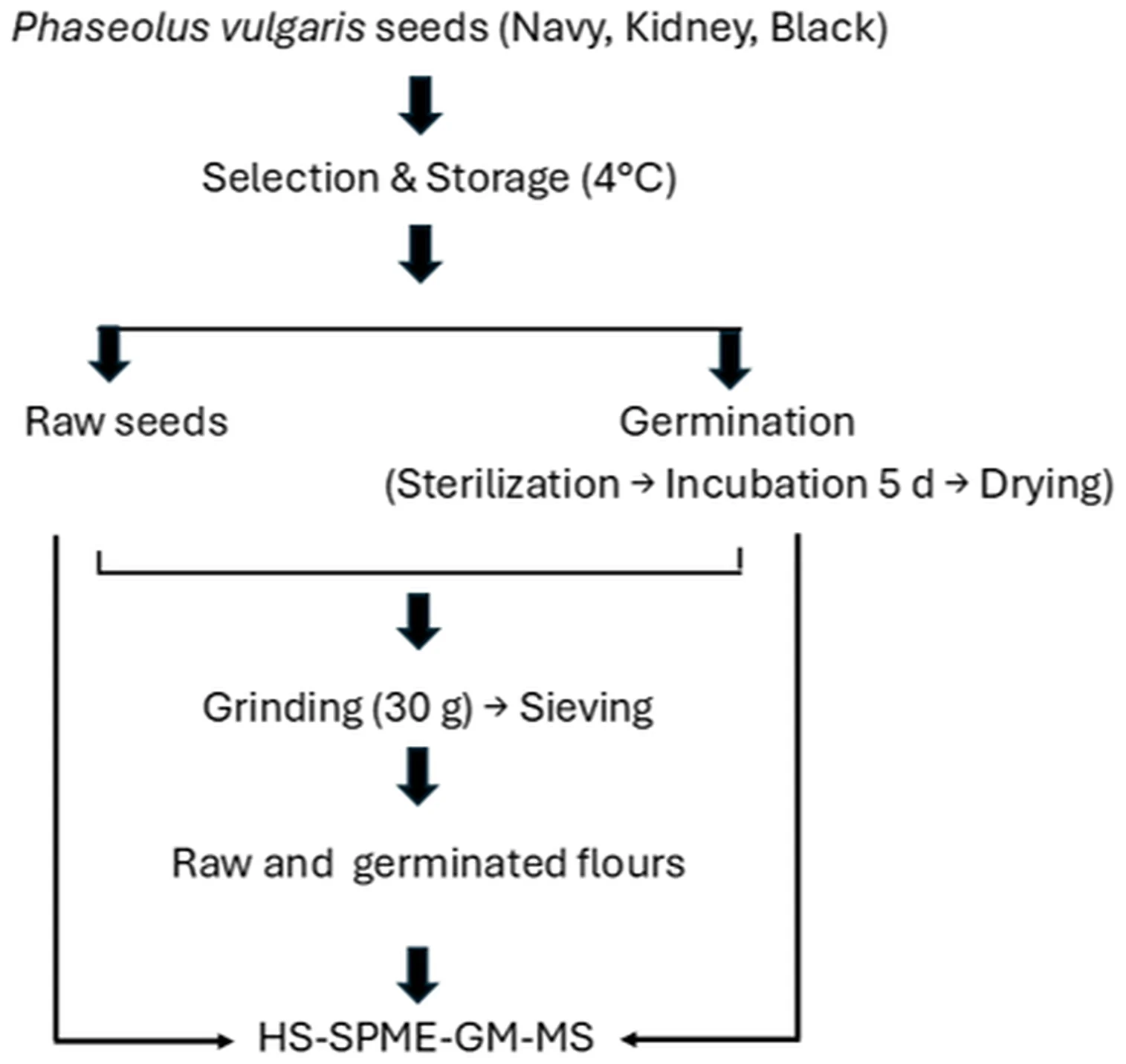
Experimental workflow for seed processing and flour preparation.

### Germination and milling processes

Seeds were surface-sterilized with 0.1% HgCl_2_ for 1 min, rinsed with sterile water, and placed on moistened filter paper in Petri dishes [2,4]. They were incubated in darkness at 20°C and 99% relative humidity for five days, then dried in a vacuum oven at 45°C for 6 h. Samples were labeled as Raw seeds (Navy-S, Black-S, Kidney-S) and Germinated seeds (G-Navy-S, G-Black-S, G-Kidney-S). Flours were prepared by grinding 30 g of each seed type, sieving through a 100-mesh screen, and labeled as Raw flours (Navy-F, Black-F, Kidney-F) and Germinated flours (G-Navy-F, G-Black-F, G-Kidney-F). All analyses were performed in triplicate.

### Headspace solid-phase micro extraction (HS-SPME)

The headspace volatiles of both whole and ground samples were collected using HS-SPME. Triplicates were analyzed for each sample type. Before sampling, each replicate was allowed to equilibrate for 30 minutes at room temperature. A solid phase microextraction fiber (Supelco, Bellefonte, PA, USA), coated with 100 μm polydimethylsiloxane (PDMS), was preconditioned as per manufacturer instructions. After exposure to the sample headspace, the fiber was retracted and immediately inserted into the GC-MS injector port for thermal desorption. Blanks were run before the first extraction and periodically during the series. Relative peak areas were used for quantitative comparisons of VOCs among samples.

### Gas Chromatography Coupled with Mass Spectrometry (GC‐MS)

GC-MS analysis was performed using an Agilent 7890B gas chromatograph coupled with an Agilent 5977B single quadrupole mass detector. An HP-5MS capillary column (30 m × 0.25 mm, 0.25 μm film thickness) was used. Operating conditions were as follows: injector temperature at 220°C, transfer line at 240°C, and oven temperature programmed from 60°C to 240°C at a rate of 3°C/min. Helium was used as the carrier gas at a constant flow rate of 1 mL/min with a 1:25 split ratio. The mass spectrometer was operated in full scan mode (scan range: 30–300 m/z, scan time: 1.0 s) [10].

### Compounds identification

Volatile compounds were identified by comparing their retention times and linear retention indices (LRIs) with those of authentic standards and *n*-alkane series. Additionally, mass spectral data were matched against commercial (e.g., NIST) and in-house libraries constructed from pure standards and known mixtures [11].

### Odor description

Odor descriptors for the identified volatile compounds were assigned based on literature sources, including the Flavornet database and previously published studies on volatile compounds in legumes and other food matrices [2–4,6]. These descriptors are provided for informational purposes to indicate the potential sensory attributes of the compounds, and do not represent direct sensory evaluation of the samples. No olfactometric analysis was performed in this study.

### Data preprocessing and statistical analysis

Prior to multivariate analysis, the dataset comprising the relative peak areas of all identified VOCs was preprocessed. To reduce the influence of large absolute differences between compounds and to emphasize relative patterns, the data were auto-scaled (mean-centered and divided by the standard deviation for each variable). No additional data transformation was applied, as the relative peak areas were already approximately normally distributed. Variables (VOCs) with zero variance (i.e., detected in fewer than two samples) were excluded from the analysis. The preprocessed dataset was used as the input for all subsequent multivariate analysis.

Principal Component Analysis (PCA) was performed on the auto-scaled VOC dataset to explore chemical differences and similarities among cultivars and treatments, identify sample groupings, and determine the key discriminant compounds. The explained variance for each component is reported. A loading plot was generated to identify the VOCs most responsible for the separation along the first two principal components.

Hierarchical cluster analysis (HCA) was performed on the same auto-scale dataset using Euclidean distance as the similarity measure. The resulting dendrogram and heatmap were used to visualize sample groupings.

The results are expressed as mean ± standard deviation (SD). One-way analysis of variance (ANOVA) followed by Duncan’s multiple range test was used to determine significant differences among cultivars within each chemical class and NTD subgroup. Differences were considered statistically significant at p < 0.05. All statistical analyses were performed using XLSTAT (version 2014).

## Results

### Effect of germination on volatile compounds in dry bean seeds

A total of 79 VOCs were identified in raw and germinated seeds of the three *Phaseolus vulgaris* cultivars. The identified compounds were grouped into seven chemical classes: monoterpene hydrocarbons (MH), oxygenated monoterpenes (OM), sesquiterpene hydrocarbons (STH), phenylpropanoids (PP), apocarotenes (AP), nitrogen- and sulfur-containing derivatives (NSD), and non-terpene derivatives (NTD).

In raw seeds, 40 VOCs were identified overall, including 18 in Navy beans-S, 28 in Kidney beans-S, and 30 in Black beans-S, accounting for 98.1%, 99.0%, and 97.3% of the total volatile emissions, respectively. In germinated seeds, 31 VOCs were detected, with 25 compounds identified in both G-Navy beans-S and G-Black beans-S, and 14 compounds in G-Kidney beans-S, representing 99.8%, 95.6%, and 97.1% of the total emissions, respectively.

As shown in Fig 2a the dominant chemical class in Navy beans-S was non-terpene derivatives (NTD; 61.4%), whereas phenylpropanoids (PP; 43.0%) predominated in Kidney beans-S, and sesquiterpene hydrocarbons (STH; 41.9%) in Black beans-S. This was followed by oxygenated monoterpenes (OM; 22.6%), NTD (19.1%), and monoterpene hydrocarbons (MH; 11.8%), respectively. The third most abundant class was PP in Navy beans-S (10.2%), NTD in Kidney beans-S (15.6%), and PP in Black beans-S (16.9%).

**Fig 2:**
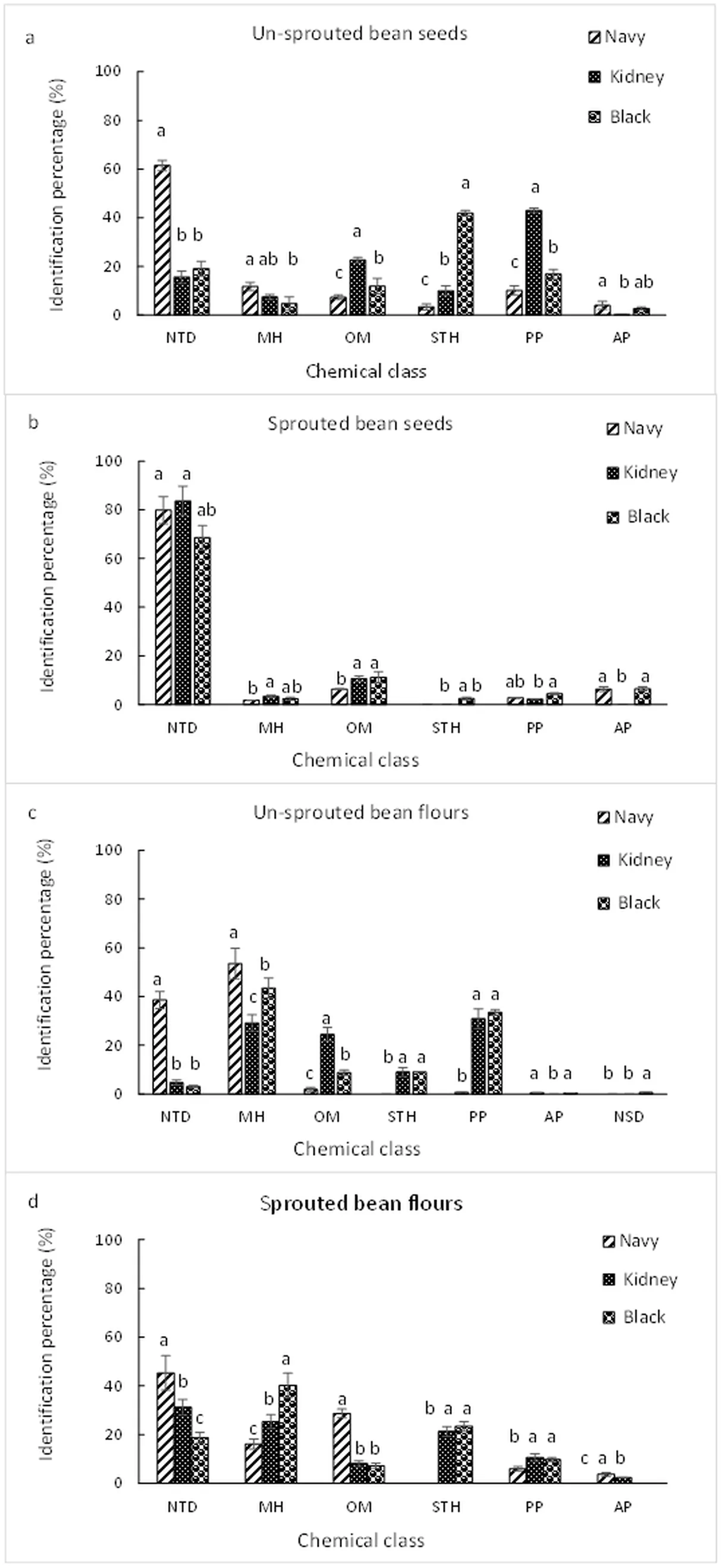
Percentages of different chemical classes emitted by un-sprouted seeds (a), sprouted seeds (b), unsprouted flours (c), and sprouted flours (d) using SPME-GC-MS. With MH: monoterpene hydrocarbons, OM: oxygenated monoterpenes, STH: sesquiterpene hydrocarbons, AP: apocarotenes, NSD: nitrogen/sulfur derivatives, and NTD: non-terpene derivatives. Different letters (a, b, b, c) above bars indicate significant differences among cultivars within the same chemical class (p < 0.05, Duncan’s multiple range test). Error bars represent standard deviation (SD) of three technical replicates.

Germination significantly altered the VOC profiles across all cultivars. In germinated seeds, NTD became the predominant chemical class, accounting for 79.8% in G-Navy beans-S, 83.6% in G-Kidney beans-S, and 68.5% in G-Black beans-S (Fig 2b). STH components were not detected in G-Navy beans-S and G-Kidney beans-S and were present only at low levels in G-Black beans-S (2.5%). This almost complete loss of sesquiterpenes, particularly striking in black beans where STH initially comprised 41.9% of raw seed volatiles, suggests extensive metabolic transformation or catabolism of these compounds during germination. PP compounds were also reduced in all germinated cultivars compared with their raw counterparts.

Thirty-nine NTD compounds were detected across all samples. Germination was associated with an overall increase in aldehydes, alkanes, ketones, alcohols, and esters, particularly in G-Black beans-S (Fig 3). Although the three cultivars shared only three VOCs across all forms: nonanal (NTD), decanal (NTD), and limonene (MH). The major compounds identified in raw seeds, including naphthalene in Navy beans-S, (*E*)-anethole in Kidney beans-S, and β-caryophyllene in Black beans-S, showed a marked decrease after germination.

**Fig 3:**
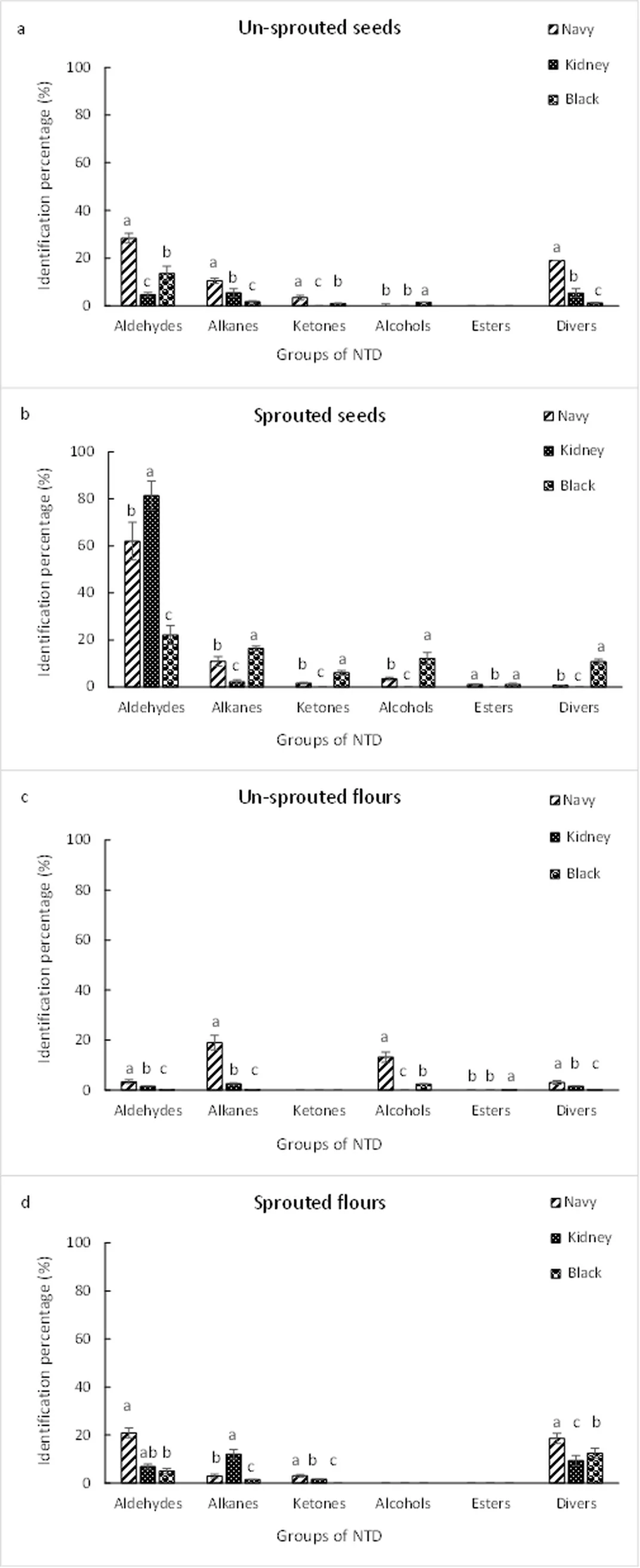
Percentages of different groups of NTD VOCs emitted by un-sprouted seeds (a), sprouted seeds (b), unsprouted flours (c), and sprouted flours (d) using SPME-GC-MS. Different letters (a, ab, b, c) above bars indicate significant differences among cultivars within the same chemical class (p < 0.05, Duncan’s multiple range test). Error bars represent standard deviation (SD) of three technical replicates.

Germination also led to the emergence of several new VOCs, including heptanal, benzaldehyde, octanal, 2-ethyl-1-hexanol, 6-methylheptyl 2-propenoate, 1-undecene, (*Z*)-3-pentadecene, 1,8-cineole, dihydrocitronellol, carvacrol, and p-cymene. In contrast, a number of VOCs detected in raw seeds, such as (*E*)-2-nonenal, p-anisaldehyde, n-undecane, naphthalene, linalool, carvone, α-humulene, ar-curcumene, and (*E*)-anethole, were no longer detected following germination (Table 1).

**Table 1:** List of VOCs identified (expressed as percentage) in raw and sprouted dry bean seeds and flours.

			Bean seeds						Bean flours						Chemical classes
			Un-sprouted			Sprouted			Un-sprouted			Sprouted			
Constituents		Odors	Navy	Kidney	Black	Navy	Kidney	Black	Navy	Kidney	Black	Navy	Kidney	Black	
1	heptanal	fatty. harsh. pungent	–	–	–	–	3.1 ± 0.9	–	–	–	–	–	–	–	NTD (aldehydes)
2	benzaldehyde	almond. burnt sugar	–	–	–	1.1 ± 0.0	2.0 ± 0.2	2.1 ± 0.3	–	–	–	–	0.6 ± 0.0	–	
3	octanal	fat. soap. lemon. green	–	–	–	2.6 ± 0.0	14.5 ± 1.8	1.3 ± 0.0	–	–	–	–	–	–	
4	nonanal	fatty. citrus-like	17.5 ± 1.3	2.0 ± 0.0	5.1 ± 0.7	38.4 ± 3.2	52.6 ± 6.4	6.9 ± 0.9	–	–	–	13.2 ± 2.0	3.1 ± 0.3	2.7 ± 0.2	
5	*(E)*-2-nonenal	fried fatty. citrus like	–	–	0.6 ± 0.0	–	–	–	–	–	–	–	–	–	
6	decanal	sweet. waxy. floral	8.4 ± 1.0	0.6 ± 0.0	5.2 ± 0.7	15.6 ± 1.9	6.3 ± 0.8	7.8 ± 1.0	3.4 ± 0.9	–	–	7.6 ± 1.1	3.1 ± 0.2	2.2 ± 0.2	
7	*(E)*-2-decenal	green	–	–	0.5 ± 0.0	–	1.0 ± 0.0	–	–	–	–	–	–	–	
8	undecanal	fatty	1.1 ± 0.5	–	0.3 ± 0.0	2.9 ± 0.7	1.0 ± 0.2	2.9 ± 0.5	–	–	–	–	–	–	
9	dodecanal	soap	–	–	0.8 ± 0.0	1.4 ± 0.3	0.9 ± 0.0	1.1 ± 0.0	–	–	–	–	–	–	
10	γ-nonalactone	–	–	0.3 ± 0.0	–	–	–	–	–	0.1 ± 0.0	–	–	–	–	
11	*p*-anisaldehyde	–	1.4 ± 0.2	1.8 ± 0.0	1.1 ± 0.0	–	–	–	–	0.5 ± 0.0	0.2 ± 0.0	–	–	–	
	*Aldehyde percentages*		28.4	4.7	13.6	62.0	81.4	22.1	3.4	1.5	0.2	20.8	6.8	4.9	
12	*n*-undecane	gasoline-like	2.5 ± 0.5	–	–	–	–	–	14.0 ± 1.1	–	–	–	1.5 ± 0.1	–	NTD (alkanes)
13	*n*-dodecane	gasoline-like	–	–	–	0.9 ± 0.0	–	–	3.3 ± 0.1	–	–	–	3.0 ± 0.1	0.8 ± 0.01	
14	3-methyldodecane	–	–	–	–	0.5 ± 0.0	–	–	–	–	–	–	–	–	
15	*n*-tridecane	gasoline-like	3.1 ± 0.3	2.6 ± 0.2	0.6 ± 0.0	1.6 ± 0.0	–	1.8 ± 0.1	1.0 ± 0.01	2.4 ± 0.01	0.2 ± 0.01	–	3.4 ± 0.2	0.7 ± 0.02	
16	*n*-tetradecane	gasoline-like	3.9 ± 0.2	2.7 ± 0.0	–	5.3 ± 0.5	2.2 ± 0.070	9.9 ± 1.08	0.7 ± 0.01	–	–	2.9 ± 0.02	4.1 ± 0.02	–	
17	3.7-dimethyldodecane	–	–	–	–	1 ± 0.0	–	–	–	–	–	–	–	–	
18	*n*-pentadecane	gasoline-like	1.1 ± 0.0	0.2 ± 0.0	1.1 ± 0.0	1.6 ± 0.1	–	4.7 ± 0.8	–	0.1 ± 0.0	–	–	–	–	
	*Alkane percentages*		10.6	5.5	1.7	10.9	2.200	16.4	19.0	2.500	0.200	2.9	12.0	1.5	
19	6-methyl-5-hepten-2-one	fruity. estery. citrusy	3.4 ± 0.8	–	1 ± 0.0	1.6 ± 0.0	–	2.8 ± 0.1	–	–	–	3.0 ± 0.1	1.6 ± 0.010	–	NTD (ketones)
20	2-nonanone	cheesy. green. herbal	–	–	–	–	–	3.2 ± 0.1	–	–	–	–	–	–	
	*Ketone percentages*		3.4	0.0	1.0	1.6	0.0	6.0	0.0	0.0	0.0	0,0	1.6	0.0	
21	2-ethyl-1-hexanol	citrus. fatty	–	–	–	0.9 ± 0.0	–	2.6 ± 0.1	–	–	–	–	–	–	NTD (alcohols)
22	1-octanol	fruty. citrus	–	–	0.9 ± 0.0	0.6 ± 0.0	–	1.5 ± 0.0	–	–	–	–	–	–	
23	1-decanol	sweat floral and fruity	–	–	–	1.0 ± 0.0	–	2.8 ± 0.1	–	–	–	–	–	–	
24	phenylethyl alcohol	–	–	–	–	–	–	–	13.2 ± 1.1	–	2.4 ± 0.0	–	–	–	
25	1-nonanol	rose orange odor	–	–	0.6 ± 0.0	1 ± 0.0	–	5.3 ± 0.0	–	–	–	–	–	–	
	*Alcohol percentages*		0.0	0.0	1.5	3.5	0.0	12.2	13.2	0.0	2.4	0.0	0.0	0.0	
26	methyl 2-ethylhexanoate	fruity. fresh	–	–	–	–	–	–	–	–	–	–	1.5 ± 0.0	–	NTD (esters)
27	6-methylheptyl 2-propenoate	–	–	–	–	1.1 ± 0.0	–	1.1 ± 0.0	–	–	–	–	–	–	
28	2-phenylethyl acetate	–	–	–	–	–	–	–	–	–	0.1 ± 0.0	–	–	–	
	*Ester percentages*		0.0	0.0	0.0	1.1	0.0	1.1	0.0	0.0	0.1	0.0	0.0	0.0	
29	2-pentyl furan	green bean. butter	–	–	–	–	–	–	–	–	–	3.1 ± 0.3	1.6 ± 0.1	2 ± 0.0	NTD (divers)
30	butyrolactone	pear–pineapple-like	–	–	–	–	–	1.5 ± 0.0	–	–	–	–	–	–	
31	3.5-octadien-2-one*	nut. crushed bug	–	–	–	–	–	–	–	–	–	–	2.4 ± 0.0	–	
32	3.5-octadien-2-one*	nut. crushed bug	–	–	–	–	–	–	–	–	–	3.3 ± 0.0	3.2 ± 0.0	1.6 ± 0.0	
33	1-undecene	gasoline-like	–	–	–	0.7 ± 0.0	–	–	–	–	–	12.2 ± 1.050	2.1 ± 0.1	8.8 ± 0.0	
34	naphthalene	tar. pungent	19 ± 1.0	5.4 ± 0.7	1.3 ± 0.1	–	–	–	3.0 ± 0.1	1.6 ± 0.6	–	–	–	–	
35	*(Z)*-3-pentadecene	–	–	–	–	–	–	9.2 ± 0.0	–	–	–	–	–	–	
36	*p*-vinylguaiacol	buckwheat aroma	–	–	–	–	–	–	–	–	0.1 ± 0.0	–	–	–	
	*Diver constituents percentages*		19,0	5.4	1.3	0.7	0,0	10.7	3,0	1.6	0.1	18.6	9.3	12.4	
37	1.8-cineole	mint. sweet	–	–	–	2.2 ± 0.1	9.0 ± 1.0	3.3 ± 0.9	–	–	–	10.7 ± 1.3	4.6 ± 1.0	–	OM
38	linalool	–	–	1.1 ± 0.0	0.7 ± 0.0	–	–	–	–	4.8 ± 0.7	2.1 ± 0.07	–	–	0.9 ± 0.6	
39	camphor	–	–	–	–	–	–	–	–	0.5 ± 0.07	0.4 ± 0.07	–	0.9 ± 0.010	–	
40	dihydrocitronellol	floral and fatty	–	–	–	0.8 ± 0.1	–	1.7 ± 0.2	–	–	–	–	–	–	
41	α-terpineol	–	–	–	–	–	–	–	–	–	0.2 ± 0.07	–	–	–	
42	carvone	spearmint. caraway	4.5 ± 0.1	14.1 ± 1.0	5.8 ± 0.8	–	–	–	–	15.1 ± 1.1	4.0 ± 0.0	–	–	–	
43	geranial	–	–	–	–	–	–	–	1.9 ± 0.0	–	–	6.4 ± 0.1	–	2.2 ± 0.1	
44	carvacrol	–	–	–	–	3.3 ± 0.2	1.6 ± 0.020	6.2 ± 0.0	–	–	–	7.0 ± 0.0	2.6 ± 0.1	2.4 ± 0.01	
45	neryl acetate	–	–	–	–	–	–	–	–	–	–	4.6 ± 0.0	–	1.7 ± 0.0	
46	4-terpineol	–	–	0.3 ± 0.0	0.5 ± 0.0	–	–	–	–	0.4 ± 0.0	0.5 ± 0.0	–	–	–	
47	cumin aldehyde	pungent. cumin-like	2.8 ± 0.0	5 ± 0.7	3.1 ± 0.0	–	–	–	–	3.1 ± 0.1	0.2 ± 0.0	–	–	–	
48	thymoquinone	–	–	1.7 ± 0.1	–	–	–	–	–	–	–	–	–	–	
49	*p*-cymen-7-ol	–	–	0.4 ± 0.0	1.8 ± 0.0	–	–	–	–	0.6 ± 0.0	–	–	–	–	
50	dihydrocarveol	–	–	–	–	–	–	–	–	–	1.2 ± 0.1	–	–	–	
51	*trans*-carveol	–	–	–	–	–	–	–	–	–	0.2 ± 0.01	–	–	–	
52	α-pinene	–	–	–	–	–	–	–	–	–	0.4 ± 0.0	–	0.7 ± 0.0	0.9 ± 0.0	MH
53	β-pinene	–	–	–	–	–	–	–	–	–	2.6 ± 0.1	–	2.1 ± 0.0	3.1 ± 0.0	
54	δ-3-carene	–	–	0.7 ± 0.0	–	–	–	–	–	4.7 ± 0.4	4.7 ± 0.1	–	1.9 ± 0.0	5.9 ± 1.0	
55	*p*-cymene	–	–	–	–	–	0.8 ± 0.0	–	–	–	–	–	4 ± 0.1	11.3 ± 1.5	
56	limonene	lemon-like	11.8 ± 1.0	6.1 ± 0.2	4.5 ± 0.1	1.8 ± 0.0	2.6 ± 0.1	2.4 ± 0.0	53.5 ± 2.7	19.6 ± 1.0	29.5 ± 1.8	16.1 ± 1.1	15.9 ± 2.1	19.0 ± 2.0	
57	γ-terpinene	herbaceous. citrus-like	–	0.7 ± 0.02	0.3 ± 0.0	–	–	–	–	3.4 ± 0.6	4.7 ± 0.1	–	–	–	
58	terpinolene	–	–	–	–	–	–	–	–	1.5 ± 0.0	1.2 ± 0.1	–	0.7 ± 0.0	–	
59	myrcene	–	–	–	–	–	–	–	–	–	0.4 ± 0.0	–	–	–	
60	δ-eIemene	–	–	–	–	–	–	–	–	0.1 ± 0.0	0.1 ± 0.0	–	–	–	STH
61	α-copaene	woody. spicy. honey	1.5 ± 0.0	1.5 ± 0.1	8.2 ± 0.5	–	–	–	–	1.2 ± 0.1	0.9 ± 0.0	–	3.9 ± 0.1	3.2 ± 0.	
62	β-elemene	–	–	–	–	–	–	–	–	0.1 ± 0.0	0.1 ± 0.0	–	–	–	
63	β-caryophyllene	woody-spicy. clove	1.9 ± 0.0	6.3 ± 0.1	31.3 ± 2.5	–	–	2.5 ± 0.0	–	5.8 ± 0.3	7.1 ± 0.7	–	11.5 ± 1.0	15.6 ± 2.1	
64	α-humulene	woody. spicy-clove	–	0.5 ± 0.0	–	–	–	–	–	0.3 ± 0.0	0.2 ± 0.0	–	–	1.5 ± 0.1	
65	*ar*-curcumene	herbaceous	–	0.8 ± 0.0	–	–	–	–	–	0.7 ± 0.0	0.4 ± 0.0	–	3.2 ± 0.1	2 ± 0.4	
66	β-selinene	–	–	0.2 ± 0.0	–	–	–	–	–	0.2 ± 0.0	0.2 ± 0.0	–	0.6 ± 0.0	1.1 ± 0.0	
67	β-bisabolene	warm. sweet-spicy	–	0.3 ± 0.1	0.7 ± 0.1	–	–	–	–	0.2 ± 0.0	–	–	1.3 ± 0.1	–	
68	β-sesquiphellandrene	woody	–	–	–	–	–	–	–	0.3 ± 0.0	–	–	0.8 ± 0.0	–	
69	α-cubebene	–	–	–	0.7 ± 0.0	–	–	–	–	0.1 ± 0.0	–	–	–	–	
70	β-cubebene	–	–	–	0.7 ± 0.0	–	–	–	–	–	–	–	–	–	
71	valencene	–	–	0.5 ± 0.0	–	–	–	–	–	–	–	–	–	–	
72	δ-cadinene	–	–	–	0.3 ± 0.001	–	–	–	–	–	–	–	–	–	
73	dihydroactinidiolide	musky. coumarin-like	–	0.2 ± 0.1	–	–	–	–	–	–	–	–	–	–	AP
74	*(E)*-geranylacetone	green. rosy. floral	4 ± 0.1	–	2.7 ± 0.0	6.4 ± 0.1	–	6.5 ± 0.8	0.5 ± 0.0	–	–	3.7 ± 0.2	2.4 ± 0.1	–	
75	methyl eugenol	–	–	2.1 ± 0.1	1.8 ± 0.1	2.8 ± 0.1	2.2 ± 0.11	4.5 ± 0.2	–	1.5 ± 0.1	–	6 ± 0.8	3 ± 0.3	1.5 ± 0.1	
76	methyl chavicole	anise. sweet	4.9 ± 0.1	2.8 ± 0.2	0.9 ± 0.0	–	–	–	–	3.4 ± 0.0	5.7 ± 1.0	–	–	2.7 ± 0.0	
77	*(E)*-anethole	anise. sweet. spicy	5.3 ± 0.5	38.1 ± 5.1	14.2 ± 1.1	–	–	–	0.7 ± 0.001	25.9 ± 1.0	27.8 ± 1.9	–	7.6 ± 0.0	5.6 ± 0.1	
78	myristicin	–	–	–	–	–	–	–	–	0.1 ± 0.0	–	–	–	–	
79	1.2-benzisothiazole	bitter almonds	–	–	–	–	–	–	–	–	0.6 ± 0.0	–	–	–	NSD
*Total identification percentages*			98.1	99.0	97.3	97.1	99.8	95.6	95.2	98.3	98.8	99.8	98.8	99.4	
* isomer not determined															

Note: Data are expressed as relative peak area percentages. The sensory relevance is inferred from literature odor descriptors.

### Effect of germination and milling on volatile compounds in bean flours

In flour samples derived from raw and germinated seeds, 56 VOCs were identified and classified into seven chemical groups: NTD (18), OM (13), STH (10), MH (8), PP (4), NSD (1), and AP (1). The total VOC identification percentages were 95.2% for Navy beans-F, 98.3% for Kidney beans-F, and 98.9% for Black beans-F, increased to 99.8% (G-Navy beans-F), 98.9% (G-Kidney beans-F), and 99.7% (G-Black beans-F). As shown in Fig 2C, MH was the dominant chemical class in Navy beans-F (53.5%) and Black beans-F (43.5%), followed by NTD (38.6%) and PP (33.5%), respectively. In contrast, Kidney beans-F exhibited a different profile, with PP as the most abundant class (30.9%), followed by MH (29.2%) and OM (24.5%). The only NSD compound identified, 1,2-benzisothiazole, was detected exclusively in Black beans-F.

Germination resulted in a marked increase in NTD compounds, which became the dominant class in G-Navy beans-F (45.3%) and G-Kidney beans-F (31.2%). The second most abundant classes were OM and MH, respectively. However, G-Black beans-F exhibited an opposite trend, with MH as the dominant class (40.2%), followed by PP (23.4%) and NTD (18.8%).

Among the NTD compounds, 12 were identified in raw flours and 13 in germinated ones. In raw samples, NTDs included alkanes (5 compounds), aldehydes (3), alcohols (1), esters (1), and miscellaneous compounds (2). Following germination and milling, the distribution shifted to alkanes (4), aldehydes (3), ketones (1), esters (1), and miscellaneous (4). Aldehyde emissions increased notably in germinated flours, particularly in G-Navy beans-F (20.8%) compared with the corresponding raw flour (3.4%). In contrast, alkanes levels decreased in G-Navy beans-F but increased in the other cultivars. Alcohols and esters were not detected in germinated flours.

Only one compound, limonene (MH), was shared among all flour samples. Its relative abundance was highest in raw flours, reaching 53.5% in Navy beans-F, and decreased markedly after germination including in G-Navy beans-F (16.1%). (*E*)-Anethole remained the dominant compound exclusively in Kidney beans-F (25.9%). Germination also led to the appearance of several new VOCs, including benzaldehyde, 6-methyl-5-hepten-2-one, methyl 2-ethylhexanoate, 3,5-octadien-2-one, 1-undecene, 1,8-cineole, and carvacrol. In contrast, a number of compounds present in raw flours, such as γ-nonalactone, p-anisaldehyde, phenylethyl alcohol, naphthalene, and carvone, were no longer detected following germination.

### Comparison of volatile constituents in raw and germinated legume seeds and flours and identification of discriminating compoundsprincipal

PCA was performed on the auto-scaled dataset of 79 VOCs identified across all raw and germinated bean seeds and flours. The first two principal components (PC1 and PC2) explained **61.11%** of the total variance, with PC1 contributing **37.60%** and PC2 contributing **23.51%**. PC1 was mainly associated with NTD, whereas PC2 was primarily correlated with AC (Fig 4).

**Fig 4:**
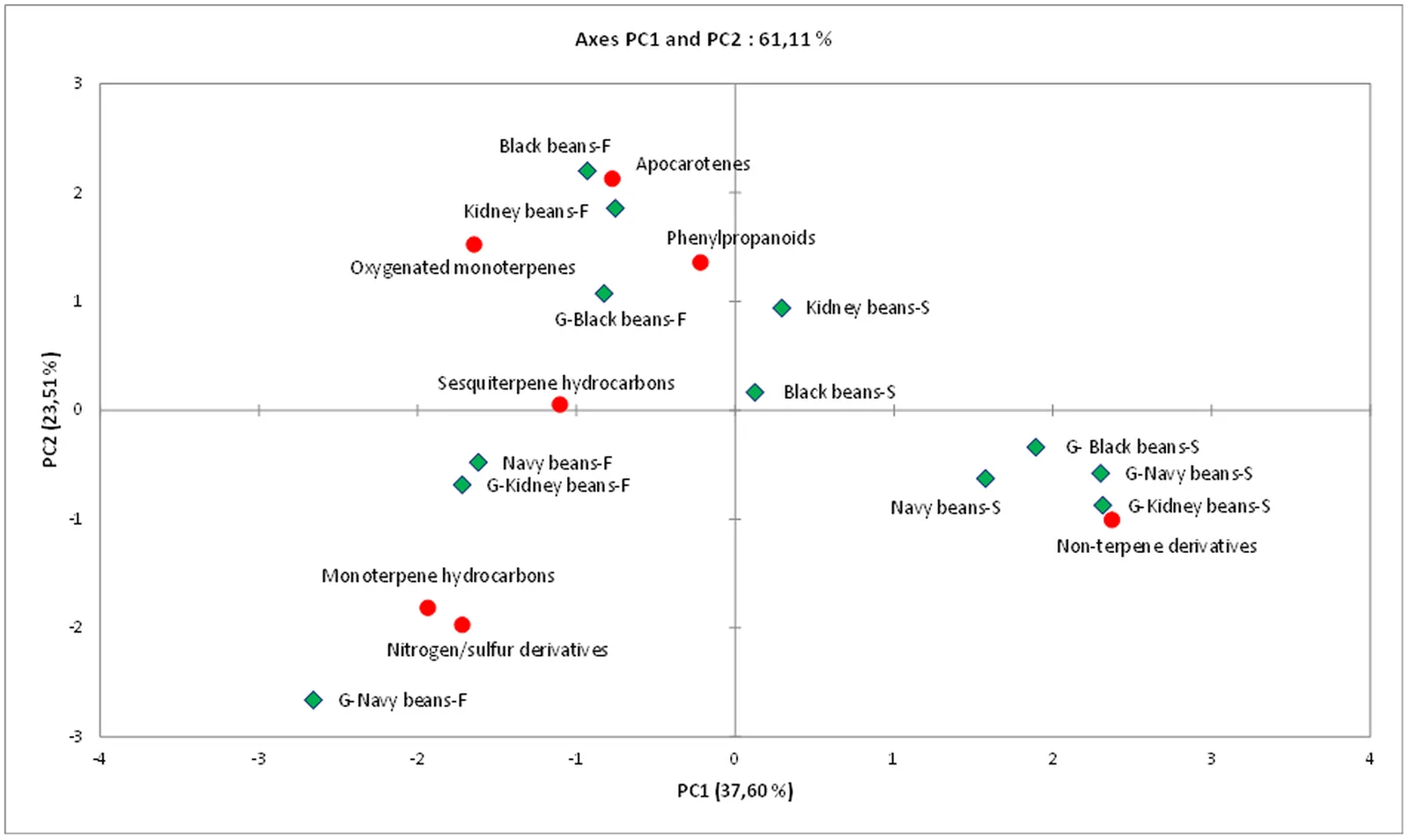
Plots from the PCA demonstrating the contribution of the volatile chemical classes and the grouping of raw (Navy beans-S, Kidney beans-S, Black beans-S, Navy beans-F, Kidney beans-F, and Black beans-F) and germinated (G-Navy beans-S, G-Kidney beans-S, G-Black beans-S, G-Navy beans-F, G-Kidney beans-F, and G-Black beans-F) samples. With: G: Germinated, S: Seeds, and F: Flours.

The PCA score plot revealed four distinct groups. Group 1, located in the upper right quadrant (positive PC1 and PC2), included Kidney beans-S and Black beans-S, which were characterized by a high content of PP. Group 2, in the lower right quadrant (positive PC1 and negative PC2), comprised Navy beans-S as well as G-Kidney beans-S, G-Black beans-S, and G-Navy beans-S, and was defined by a dominance of NTD. Group 3, situated in the lower left quadrant (negative PC1 and PC2), consisted of Navy beans-F, G-Kidney beans-F, and G-Navy beans-F, which were distinguished by higher levels of MH and NSD. Group 4, located in the upper left quadrant (negative PC1 and positive PC2), included Black beans-F, Kidney beans-F, and G-Black beans-F, and was characterized by higher contributions of OM, AP, and PP (Fig 4). Overall, the distribution of samples across the PCA space highlighted clear chemical differentiation according to both cultivar and processing stage (raw versus germinated, seed versus flour), suggesting that specific volatile compounds may act as discriminant markers contributing to distinct aroma profiles.

### Correlations among the chemical classes

Pearson correlation analysis was performed to assess relationships among the seven chemical classes of VOCs identified in raw and germinated dry bean seeds and flours. The results are presented in Table 2.

**Table 2:** Pearson’s correlation coefficients (r) among the identified chemical classes of raw and germinated dry bean seeds and flours.

Variables	MH	OM	STH	PP	AC	NSD	NTD
MH	1						
OM	0.370	1					
STH	0.109	-0.104	1				
PP	-0.156	0.083	0.111	1			
AC	-0.239	0.392	0.155	-0.092	1		
NSD	0.794	0.012	0.287	-0.170	-0.024	1	
NTD	-0.541	-0.733	-0.473	-0.321	-0.409	-0.416	1

Significant positive correlations were observed between MH and NSD (r = 0.794), MH and OM (r = 0.370), and OM and AP (r = 0.392). These positive associations suggest that increases in one chemical class tend to coincide with increases in the others, indicating possible co-occurrence patterns or shared biosynthetic or metabolic pathways.

In contrast, negative correlations were observed between STH and OM (r = –0.104), NSD and PP (r = –0.170), and NTD and multiple classes, particularly MH (r = –0.541), OM (r = –0.733), and NSD (r = –0.416). These inverse relationships indicate that increases in NTD compounds are generally associated with decreases in terpene classes such as MH and OM. This pattern is consistent with the effects of germination, during which NTD compounds became predominant while terpene-related classes (MH, OM, and STH) declined.

Overall, these correlation patterns reflect marked shifts in VOC chemical profiles induced by germination and milling, highlighting clear reorganization of volatile composition across processing stages. They also suggest the potential for targeted modulation of aroma characteristics through controlled processing conditions.

### Hierarchical cluster analysis

To further investigate the effects of sprouting and milling on dry bean seeds, hierarchical cluster analysis was performed based on the relative abundance of VOC chemical classes, and the results are presented in the heatmap shown in Fig 5. The analysis revealed three main clusters. Cluster 1 (C1) included Navy beans-S, G-Navy beans-S, G-Black beans-S, and G-Kidney beans-S, and was characterized by relatively high levels of NTD, reflecting the impact of germination on VOC composition. Cluster 2 (C2) grouped Black beans-S and Kidney beans-S, which were associated with higher proportions of PP and STH, typical of raw seed profiles. Cluster 3 (C3) comprised all flour samples, including Black beans-F, Kidney beans-F, Navy beans-F, G-Navy beans-F, G-Black beans-F, and G-Kidney beans-F, and was defined by elevated levels of MH and, in some cases, OM, indicating a distinct VOC pattern associated with milling.

**Fig 5:**
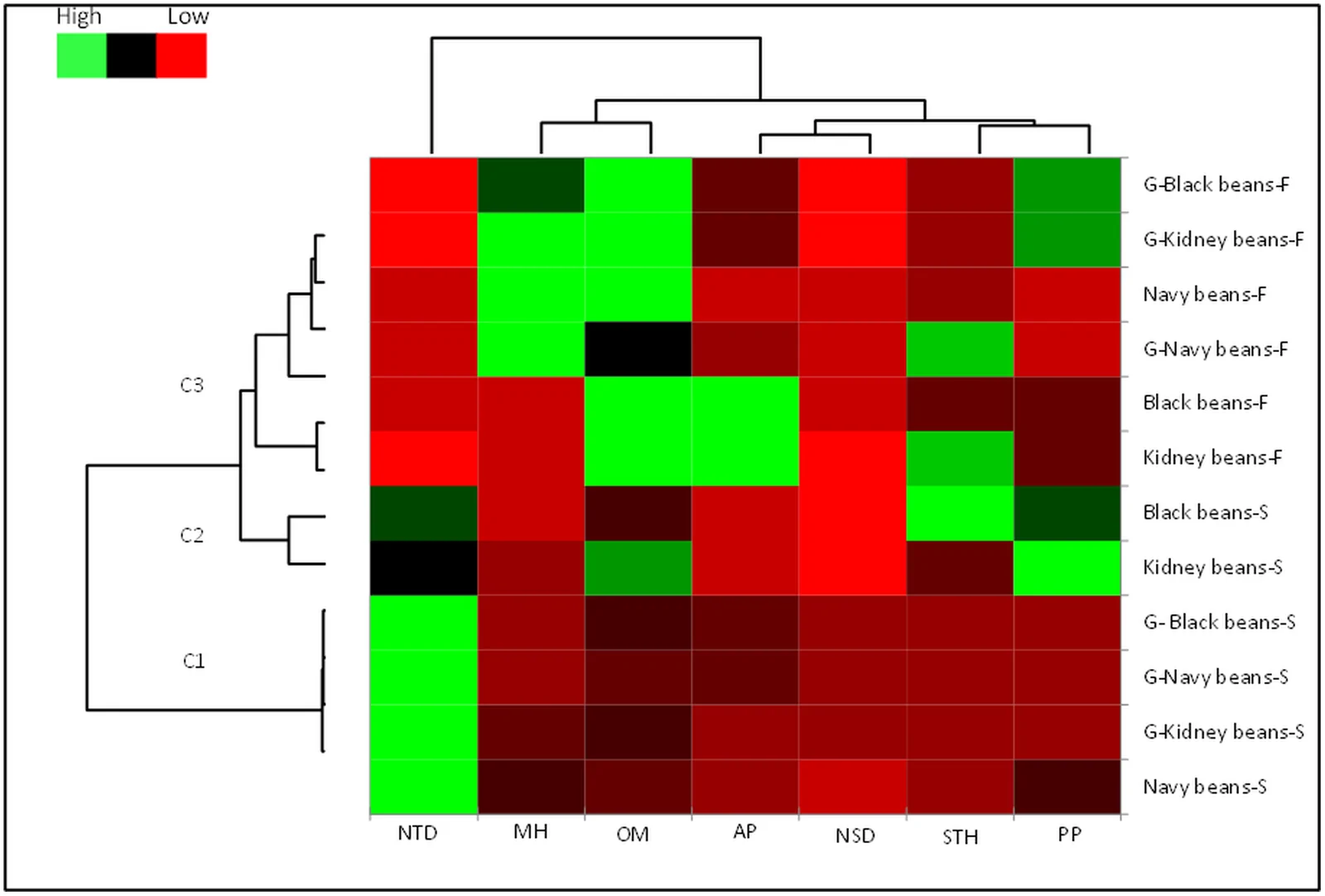
Heatmap based on Euclidean distances using different chemical classes of of raw (Navy beans-S, Kidney beans-S, Black beans-S, Navy beans-F, Kidney beans-F, and Black beans-F) and germinated (G-Navy beans-S, G-Kidney beans-S, G-Black beans-S, G-Navy beans-F, G-Kidney beans-F, and G-Black beans-F) samples. The different clusters C1, C2, and C3 correspond to the individualized groups. With: G: Germinated, S: Seeds, and F: Flours.

The heatmap color scale illustrates these differences, with red indicating lower abundance, black intermediate levels, and green higher abundance. For example, NTD levels ranged from 83.6% (G-Kidney-S, green/high) to 0.2% (Black-F, red/low), while MH levels ranged from 53.5% (Navy-F, green/high) to 1.8% (G-Navy-S, red/low). Overall, the clustering clearly differentiates seeds from flours as well as raw from germinated samples, confirming the strong influence of both germination and milling on the VOC profiles of dry beans.

## Discussion

Germination is a physiological process that enhances the nutritional and functional quality of legumes through extensive metabolic reprogramming [12,13]. Upon imbibition, seed metabolism is reactivated, leading to the synthesis of enzymes involved in carbohydrate, protein, and lipid mobilization, which support embryo growth and development [14]. This activation includes hydrolytic enzymes as well as key regulatory enzymes of secondary metabolism, particularly phenylalanine ammonia-lyase (PAL), which initiates the phenylpropanoid pathway responsible for the biosynthesis of phenolic and flavonoid compounds. Consequently, germination promotes both de novo synthesis and the release of bound phenolics previously associated with cell wall components, thereby increasing their extractability and bioavailability. Structural modifications of the seed matrix induced by enzymatic hydrolysis further facilitate the liberation of phytochemicals and enhance metabolic flux toward antioxidant-related compounds, often resulting in increased antioxidant capacity and reduced antinutritional factors, depending on species and germination conditions [15].

In parallel, germination markedly alters the VOC profile through coordinated metabolic and structural transformations [12]. Lipid oxidation via lipoxygenase-dependent pathways generates volatile aldehydes, ketones, and alcohols, while amino acid catabolism contributes nitrogen- and sulfur-containing volatiles, thereby increasing chemical diversity. Secondary metabolic pathways, including phenylpropanoid metabolism and carotenoid degradation, are also activated, leading to the formation of oxygenated compounds such as phenolic derivatives and apocarotenoids [15]. At the same time, structural breakdown of the seed matrix enhances the release of bound or matrix-associated volatiles [12], whereas storage-related hydrocarbons and terpenes generally decrease due to metabolic conversion, volatilization, or utilization in respiration [15].

HS-SPME/GC-MS is a well-established and reliable technique for evaluating the effects of sprouting and milling on the VOC composition in legumes and other food matrices [16–19]. In the present study, germination resulted in a marked increase in NTD compounds, particularly aldehydes, across all cultivars and sample types, with the most pronounced increase observed in *G-Kidney beans-S* (Table 1; Fig 2 and 3).

These findings are consistent with previous studies reporting that prolonged germination increases beany off-flavors in faba bean and lentil seeds [20–22]. Aldehydes play a key role in this context, which is important due to their low odor thresholds and strong impact on the overall aroma profile of legumes. In the present study, several aldehydes including heptanal (fatty, citrusy, rancid), benzaldehyde (almond-like, burnt sugar), and octanal (soapy, lemony, green) were identified only in sprouted seeds, not in the raw versions [23]. Additionally, other aldehydes, such as nonanal (fatty, citrus) and decanal (sweet, floral) were present at higher relative abundances in germinated seeds and the corresponding flours [24].

Lipid oxidation is a major source of aldehydes and significantly contributes to food aroma because our noses are extremely sensitive to them and they have very low odor thresholds. For example, the aldehyde hexanal smells “grassy” at just 0.0045 parts per billion (ppb), and (*E*, *E*)-2,4-decadienal smells “fatty” or “roasted” at only 0.00007 ppb (Belitz et al., 2009). In contrast, alkanes have very high odor thresholds [25]. The alkane pentadecane, for instance, needs to be present at 3000 ppb before you can smell it [26]. That means an alkane must be millions of times more concentrated than an aldehyde to have any smell at all. Alkanes also produce only weak, chemical-like odors, while aldehydes create distinct, recognizable smells like grassy, green, fatty, or roasted [6]. Therefore, although alkanes are often more abundant in oxidized foods, they have almost no impact on aroma. Aldehydes, despite being less abundant, are the main contributors to food aroma because they are so potent [25].

It should be noted that these compounds are known from the literature to have characteristic odor descriptors (e.g., fatty, green, citrusy); however, their actual sensory contribution in the present samples cannot be determined without quantitative analysis. Our results therefore represent a chemical profile of volatile organic compounds (VOCs) rather than a direct assessment of sensory impact*.*

This study clearly showed that alkanes were the second most abundant VOC group in raw and germinated seeds and their levels increased significantly in some cases (*G-Black beans-S*), which matches earlier research showing that lipid peroxidation produces many alkanes [16,20]. However, high abundance does not mean strong aroma.

Our experiment also found that alcohols increased in germinated seeds, likely because of dehydrogenase enzymes, which convert aldehydes into alcohols. Some alcohols can contribute green or grassy notes to flavor [27]. However, in legumes, alcohols are generally considered undesirable because they often create heavy, solvent-like, or painty off-flavors rather than fresh, pleasant aromas [17]. Compared to aldehydes, alcohols have higher odor thresholds, meaning they are less potent. So, while germination increases alcohols through dehydrogenase activity, this is often a negative change for legume flavor, moving the profile away from fresh and toward undesirable notes.

In contrast to germination, milling reduced the levels of both aldehydes and alkanes. This makes sense because milling removes the outer layers of seeds, which are rich in lipids and enzymes that drive oxidation. A similar trend has been seen in brown rice, where more intensive milling lowered the concentrations of lipid-derived volatile compounds [28]. The oxidative breakdown of unsaturated fatty acids and amino acids produces aldehydes such as octanal, nonanal, and decanal, and we found that all three were more abundant in germinated seeds than in raw ones [20]. Germination activates enzymes and exposes more lipids to oxygen, increasing aldehyde formation. Milling does the opposite: it physically removes the lipid-rich layers, reducing the raw materials needed to produce these volatile compounds. So, germination boosts aldehyde levels (which strongly affect aroma), while milling lowers them by stripping away the source of those flavors.

Regarding terpene compounds like MH, OM, and STH that often contribute floral, piney, or citrus notes to foods. In our case, germination reduced total terpene content in seeds, while milling increased MH levels, especially in flour samples. This is interesting because it partially differs from earlier work on faba beans, where flour and whole seeds showed similar terpene patterns [21]. The changes likely happen for different reasons: germination may activate enzymes that break down terpenes or convert them into other compounds, while milling physically disrupts seed tissues, possibly releasing terpenes that were previously bound or hidden inside cells. The increase in MH after milling could also come from mechanical shearing or heat generated during grinding, which might liberate or form new terpenes. Unlike aldehydes, which clearly signal lipid oxidation, terpenes respond more variably to processing, their levels can go up or down depending on the seed type, the specific terpene, and how the treatment affects plant cell structures and enzyme activity [4,6].

Unlike NTD compounds (which include aldehydes, alkanes, and alcohols that come mainly from lipid oxidation), terpenes are not produced by random breakdown of fats. Instead, plants make them through specific, dedicated metabolic pathways [29]. Among all the terpenes, limonene was the most dominant and was found in every sample. Limonene has a pleasant citrus aroma, and it contributes positively to legume flavor, unlike many lipid oxidation products (like heavy alcohols or rancid aldehydes) that are considered undesirable. Previous work on common beans (*Phaseolus vulgaris*) also identified limonene as a key volatile compound [16].

In this experiment, the levels of monoterpenes like limonene and *p*-cymene are really different from faba beans reports. These compounds are usually a lot lower in faba beans [21,22]. This difference probably happens because of both biological and methodological factors. Biologically, *dry beans* have distinct terpene synthase genes compared to other legumes. In addition, growing conditions strongly influence terpene production [30]. Methodologically, our use of a non-polar PDMS fiber preferentially extracts monoterpene hydrocarbons, whereas studies on faba beans typically used mixed-phase fibers (e.g., CAR/PDMS) that favor polar volatiles [20].

A more striking change was that STH almost completely disappeared after germination. In black beans, STH dropped from 41.9% of the volatile profile in raw seeds to only 2.5% in germinated ones. This dramatic decrease may be explained by several factors. First, germination activates oxidative enzymes, such as lipoxygenases and cytochrome P450s, which can convert sesquiterpenes into less volatile oxygenated compounds like alcohols, ketones, or acids that are not detected as free volatiles [30,31]. Second, the breakdown of cell walls during germination may release bound terpenes from storage sites, making them more susceptible to further transformation or irreversible binding to other seed components [12]. Third, the metabolic reactivation during germination may redirect terpenoid precursors toward the synthesis of other compounds, such as defence-related metabolites [15,30].

It is important to note that while terpenes could theoretically be used for energy, there is little evidence that they serve as significant energy sources during germination, as starch, proteins, and lipids are the primary reserves for the developing embryo [12,14]. The fact that black beans retained a small amount of STH (2.5%) while navy and kidney beans lost them completely suggests that terpene metabolism during germination varies among cultivars [32].

The oxidative stability of lipids during processing is a critical factor influencing the formation of volatile compounds, particularly aldehydes, which are potent aroma contributors [25]. As demonstrated by Frankel and Huang [33], blending polyunsaturated vegetable oils with high-oleic sunflower oil improves oxidative stability by reducing the susceptibility of lipids to peroxidation. This principle is relevant to the present study, as the marked increase in aldehyde levels observed during germination reflects enhanced lipid oxidation due to the activation of lipoxygenase enzymes and increased exposure of unsaturated fatty acids to oxygen [33]. The differential responses among cultivars may therefore be partly attributed to variations in their inherent fatty acid composition and oxidative stability. Understanding these relationships is essential for developing processing strategies that optimize the flavor quality of legume-based products.

## Conclusion

This study comprehensively evaluated the effects of germination and milling on the volatile organic compound profiles of three *Phaseolus vulgaris* cultivars using HS-SPME-GC-MS combined with multivariate analysis. Germination significantly increased non-terpene derivatives, particularly aldehydes derived from lipid oxidation, while decreasing terpene-based compounds and phenylpropanoids. This shift indicates that germination activates lipid metabolism and enhances the formation of volatile compounds associated with both positive and negative aroma notes. In contrast to germination, milling reduced aldehyde and alkane levels, likely due to the physical removal of lipid-rich outer seed layers. However, milling increased monoterpene hydrocarbons, especially in flour samples, suggesting that mechanical disruption releases or generates new terpenes. Multivariate analysis and hierarchical clustering clearly distinguished samples by cultivar, treatment (raw vs. germinated), and form (seed vs. flour). NTDs were the key discriminant compounds for germinated samples, while MH and PP characterized raw flours and raw seeds, respectively.

The almost complete loss of sesquiterpene hydrocarbons during germination in black beans reveals extensive metabolic transformation of terpenoid pools. These findings chemically characterize germination and milling-induced VOC changes in three *Phaseolus vulgaris* cultivars. However, it should be noted that the drying of sprouted seeds at 45°C for 6 h may have contributed to aldehyde formation through thermal oxidation, and thus the observed increases in aldehydes cannot be attributed exclusively to enzymatic lipid oxidation during germination. *Further research incorporating gas chromatography-olfactometry and consumer sensory testing is needed to translate these chemical findings into flavor optimization strategies.*

## Supporting information

S1 File Supplement data. (XLSX)

## Abbreviations

AC: apocarotenes
MH: monoterpene hydrocarbons
OM: oxygenated monoterpenes
STH: sesquiterpene hydrocarbons
NSD: nitrogen- and sulfur-containing derivatives
NTD: non-terpene derivatives
PP: phenylpropanoids
VOCs: volatiles organic compounds
